# Origin, Generation, and Destination Country Context: Employment Changes and Childbearing Among Female Immigrants and Their Descendants in the UK, France, and Germany

**DOI:** 10.1007/s10680-025-09750-w

**Published:** 2025-10-13

**Authors:** Júlia Mikolai, Hill Kulu, Isaure Delaporte, Chia Liu

**Affiliations:** 1 School of Geography and Sustainable Development, University of St Andrews, St Andrews, UK; 2 Research Department, International Labour Organisation, Geneva, Switzerland

**Keywords:** Employment, Childbearing, Immigrants, Descendants, Event history analysis, Cross-national comparison

## Abstract

This study investigates the link between childbearing and employment changes of female immigrants and their descendants in three European countries: the UK, France, and Germany. Although childbearing significantly influences female labour force participation, the interrelationship between fertility and employment changes among migrant populations is poorly understood. We use event history models to study employment entry and exit by migration background and parity. Mothers are less likely to enter and more likely to exit employment than childless women among native women, immigrants, and their descendants. The largest differences in employment entry and exit are observed between migrant origin groups and generations, and between destination countries. European and Western immigrants are more likely to (re-)enter and less likely to exit employment than those from non-European countries. The descendants of immigrants have higher employment levels than immigrants and the differences compared to natives are smaller, but they persist, particularly among those of non-European descent. We also observe some differences across countries: mothers are the most likely to exit employment in Germany and the least likely in France. Our study highlights the importance of work–family reconciliation and immigration policies for reducing labour market disadvantage among mothers overall and particularly among immigrants and their descendants.

## Introduction

Labour market participation is one of the key dimensions of immigrant integration. A large literature has focused on the employment and labour market outcomes of immigrants and their descendants across Europe. These studies found that immigrants and their descendants experience labour market disadvantage; they have lower wages and employment levels than natives across Europe (e.g. Algan et al., 2010; Heath et al., 2008; Meurs et al., 2006; Rendall et al., 2010). Differences in the experiences of natives and immigrant/minority populations are especially large among non-European/non-Western groups.

Female immigrants and their descendants may experience higher labour market disadvantage than native women due to their differential childbearing trajectories. Immigrants and their descendants from culturally and geographically distant traditional sending countries^1^ tend to have larger families than native women (e.g. Kulu et al., 2017, 2024). However, it remains unclear to what extent the employment trajectories of immigrants and their descendants are related to their childbearing trajectories. Among majority populations, having children has a well-known negative influence on women’s labour market participation (Matysiak & Vignoli, 2008). However, only a handful of studies investigated whether and how the employment trajectories of immigrants (e.g. Liu & Kulu, 2022; Mikolai & Kulu, 2025; Vidal-Coso, 2019) or their descendants (e.g. Delaporte & Kulu, 2024; Holland & de Valk, 2017; Maes et al., 2021; Mikolai & Kulu, 2022a) are related to their childbearing and how this relationship varies across different origin groups.

Even fewer studies focused on the fertility–employment nexus among both immigrants and descendants (e.g. Kil et al., 2018; Wood & Neels, 2017). Studying both the first (immigrants) and second (descendants) generation^2^ is essential to understand whether and how immigrants’ experiences change across generations. Due to the lack of studies that have focused on employment and childbearing among both immigrants and their descendants, we do not know whether differences between immigrant and native women’s experiences persist or decrease across migrant generations.

Women’s fertility and labour market experiences are likely shaped by the institutional context of work–family and immigration policies. Although cross-national studies are available on immigrants’ (e.g. Kogan, 2006; Rendall et al., 2010) or their descendants’ employment (e.g. Algan et al., 2010; Heath et al., 2008) or childbearing (e.g. Kulu et al., 2017), there is a paucity of cross-national evidence on the link between childbearing and employment across two migrant generations.

We investigate the interrelationship between childbearing and employment among female immigrants and their female descendants as well as those of native women in the UK, France, and Germany, the three largest European immigration countries. We offer several novel contributions to the literature. First, we focus on (first and second) employment entries as well as employment exit by parity. This allows us to explore women’s employment and childbearing trajectories in tandem, which no previous study has done. Second, we study childbearing and employment changes among both immigrants and their descendants from different origin groups. This allows us to understand whether and how (a) employment and childbearing are differentially linked among women from different origin groups and (b) the interplay between childbearing and employment changes across migrant generations. This is important to understand the role of adaptation and socialisation for childbearing and labour market disadvantage across migrant generations. Third, we compare the experiences of immigrants and their descendants across the UK, France, and Germany; three countries characterised by different welfare and immigration regimes, and work–family policies. This allows us to investigate how the country context shapes different groups of immigrants’ and their descendants’ childbearing and employment.

## Employment Among Immigrants and Their Descendants

Immigrant women have traditionally been tied or family migrants, implying that they were often either not allowed to work or faced challenges on the labour market such as discrimination, lack of recognition of their qualifications, or language issues (Röder et al., 2018). Evidence shows that compared to natives, immigrants are disadvantaged on the labour market across Europe. They have lower labour market participation (Dustmann et al., 2003), employment (Blackaby et al., 1997; Dustmann & Fabbri, 2003; Wheatley Price, 2001), and earnings (Chiswick, 1980; Dustmann et al., 2003). This disadvantage is largely explained by immigrants’ lower human capital, education, socio-economic status, and their demographic characteristics at the time of migration (Borjas, 2013; Dustmann et al., 2003).

Immigrants from different countries have different experiences. Those from non-European countries are at a particular disadvantage even after adjusting for socio-economic differences between natives and immigrants. Potential reasons for this include the lack of language fluency, destination country-specific human capital, transferable skills and qualifications, weaker social networks, and discrimination (Dustmann & Fabbri, 2003; Dustmann et al., 2003; Rendall et al., 2010; Salikutluk et al., 2020). For example, in the UK,^3^ Black African, Pakistani, and Bangladeshi immigrants have much lower employment rates than white UK-born individuals or white immigrants (Blackaby et al., 1997; Dustmann & Fabbri, 2003; Dustmann et al., 2003). Similarly, in France, women from North Africa have a significantly higher risk of unemployment than native women (Meurs & Pailhé, 2008). Finally, in Germany, Turkish women are the least likely to engage in the labour market (Guveli & Spierings, 2022; Salikutluk et al., 2020). Evidence from other Western European countries shows similar trends (Kogan, 2006; Rendall et al., 2010).

The descendants of immigrants are born in the destination countries to immigrant parents. As they are educated and socialised in the destination countries, their values, preferences, and behaviours are expected to be similar to those of natives according to the assimilation or integration hypothesis (Alba & Nee, 2003). This suggests that the second generation should experience less labour market disadvantage than immigrants (Heath & Cheung, 2007). At the same time, the second-generation grows up in a family of immigrants (Adsera & Ferrer, 2015; Kulu et al., 2019), implying that some groups may be socialised into the norms, values, preferences, and behaviours common in their parents’ country of origin (minority subculture hypothesis (Goldscheider & Uhlenberg, 1969; Portes & Zhou, 1993)) whilst other groups may grow up surrounded by norms, preferences, and behaviours common among majority populations (Kulu et al., 2019). Additionally, some groups of descendants may face discrimination (minority group status hypothesis (Milewski, 2010a)), which may lead to reduced labour market opportunities. This may especially be the case among women who may choose to follow the ‘motherhood track’ (Kulu et al., 2019) in anticipation of limited labour market prospects (Heath et al., 2008).

Evidence shows that the descendants of immigrants experience labour market disadvantage across Europe (e.g. Clark & Drinkwater, 2010; Clark & Ochmann, 2022; Meurs et al., 2006; Piton & Rycx, 2021; Zwysen & Demireva, 2020). For example, most descendant groups had significantly lower net hourly wages and/or levels of employment than natives in France, Germany, and the UK (Algan et al., 2010). A study that reviewed evidence from Austria, Belgium, Britain, France, Germany, the Netherlands, and Sweden also found that descendants of immigrants from economically less developed countries were more likely to be unemployed than native women (Heath et al., 2008). These studies do not support the assimilation or integration hypothesis, but the data do not allow distinguishing whether differences persist due to the minority subculture or minority group status hypothesis or discrimination, or potentially a combination of these.

## Childbearing and Employment Among Immigrants and Their Descendants

Immigrant women from different origin countries have different childbearing patterns. Non-western immigrants tend to have children earlier and have larger families (Kulu et al., 2017), whereas European or Western immigrants have similar childbearing patterns to native women (e.g. Milewski, 2007, 2010b). This means that many non-European immigrant women not only have limited labour market opportunities and lower wages but also care for several children. Given the costs of childcare and limited earnings opportunities, many immigrants may not be able to afford childcare or limited career opportunities and low wages among immigrant women may not provide financial incentives to work (Röder et al., 2018). This position, coupled with conservative values in some non-European origin countries, may mean that some groups of immigrant women are unlikely to enter employment, but if they do, they are likely to exit the labour force and do not re-enter employment after becoming mothers. Thus, we expect that non-European immigrant women’s employment will be more affected by childbearing than that of European immigrant women. In other words, among non-European immigrant women, the change in employment (re-)entry and exit due to childbearing will be larger than among European immigrant women (H1a; origin hypothesis among immigrants).

The available evidence on the employment–fertility nexus among immigrants is limited. In the UK, motherhood is coupled with heterogeneous employment patterns (Mikolai & Kulu, 2025). South Asian women had early and universal marriage and childbearing coupled with economic inactivity (Mikolai & Kulu, 2025). In contrast, European and Western immigrant women started relationship and family formation later and were in education or full-time employment (Mikolai & Kulu, 2025). In Germany, immigrant mothers were less likely to work full-time, more likely to exit the labour market, and less likely to re-enter employment than childless immigrant women. Women from former Yugoslavia, the rest of Europe, and the former Soviet Union were more attached to the labour market than other groups (Liu & Kulu, 2022). In Belgium, the economic activity and employment levels of immigrant women from Europe, Morocco, and Turkey have decreased more than among native women after becoming a mother (Kil et al., 2018). Finally, in Switzerland, working hours and employment levels of immigrant and native women declined substantially following childbirth (Vidal-Coso, 2019).

Overall, the fertility of second-generation women is similar to that of native women (Kulu & Hannemann, 2016; Kulu et al., 2017) although some descendant groups have larger families than native women (e.g. Kulu & Hannemann, 2016; Kulu et al., 2017; Mikolai & Kulu, 2022b; Milewski, 2007; Pailhé, 2017). This means that labour market disadvantage may be coupled with raising larger families for many second-generation women. Based on the assimilation hypothesis, we would expect that across migrant generations the interrelationship between employment and childbearing would become more similar to that observed among native women. However, the magnitude of this change is likely to vary across origin groups. Thus, we expect that second-generation women’s employment (re-)entry and exit levels will be more similar to those of native women than to immigrant women from the same origin (H2: generation hypothesis). This will especially be the case among the descendants of European immigrants, and we expect to see more differences (i.e. less change across generations) among the descendants of non-European immigrants (H1b: origin hypothesis among descendants).

Only a handful of studies have examined how childbearing influences female descendants’ employment. For example, in Germany, Sweden, the Netherlands, and France both native and second-generation Turkish mothers were less likely to participate in the labour force than childless women (Holland & de Valk, 2017). In Belgium, the economic activity and employment levels of the second generation decreased more than those of native women following childbearing (Kil et al., 2018). Additionally, the lower maternal employment levels and working hours of second-generation (especially Moroccan or Turkish) women were explained by their lower employment rates and working hours already prior to childbirth compared to native women (Maes et al., 2021). All groups of second-generation mothers and native mothers in France were more likely to exit employment than childless native women (Delaporte & Kulu, 2024). Turkish descendants were the least likely to enter and most likely to exit employment once becoming mothers. Finally, in the UK, European/Western descendants had similar employment and family trajectories to native women (Mikolai & Kulu, 2022a). Although Caribbean descendants had different family patterns, their employment outcomes were similar to those of native women. Female South Asian descendants had conservative family formation patterns and low labour market attachment.

## The Cross-National Context

### Migration History

In the UK, immigrants have arrived from former colonies. In the 1950s and 1960s, labour migration from the New Commonwealth (e.g. Caribbean, India, Pakistan, Bangladesh) was dominant, followed by family reunification (Dale & Ahmed, 2011; Dubuc, 2012). Starting in the 1970s, immigration from sub-Saharan Africa became common (Coleman & Dubuc, 2010; Dubuc, 2012) due to family reunification, asylum seeking, and better education and employment opportunities (Barou et al., 2012; Spaan & van Moppes, 2006). During the 1990s, the largest source of immigration was family reunification and asylum seeking (Sainsbury, 2012). More recently, the UK has received immigrants from the European Union (especially Poland) and China (e.g. Robards & Berrington, 2016).

In Germany, immigrants have arrived for work, family reunification, or political refuge. As part of Germany’s rebuilding efforts during the 1960s and 1970s, labour migrants arrived particularly to West Germany from Turkey, Italy, Greece, and Yugoslavia, countries that Germany has signed recruitment treaties with (Münz & Ulrich, 1998). Many of these labour migrants settled permanently, and this wave of migration was quickly followed by family reunification (Bade & Weiner, 1997). Following the fall of the Iron Curtain, many Ethnic Germans (*Aussiedlers*) from former Soviet countries repatriated to Germany; they were granted a special status as individuals of German descent which entitled them to German citizenship and integration support upon arrival (Bade & Weiner, 1997; The Expert Council on Integration & Migration, 2022). From 2004, the Eastern Enlargement of the European Union led to substantial increases in migration from European countries to Germany (Elsner & Zimmermann, 2016). Immigrants from Turkey, Syria, Russia, Poland, Ukraine, and Kazakhstan form the biggest groups in Germany (DESTATIS, 2024).

In France, immigrants have arrived from former colonies of Vietnam, Algeria, and Sub-Saharan African and Asian countries between 1945 and 1974 mostly as migrant workers (Algan et al., 2010). After 1974, family reunification became the main reason for migration. Asylum seeking has also become more common after the 1980s (Migration Policy Institute, 2004).

### Work–Family Policies

Women’s labour market outcomes are influenced by work–family policies. The three countries in this study represent different policy regimes regarding the gendered character of the welfare regime and the support available to combine paid work and childcare. The UK views both women and men as invested in employment but has limited support for childcare, which is provided by the market and is costly (Kowalewska, 2023; Misra et al., 2007). Female employment tends to be low and many work part-time (Kowalewska, 2023). Employed women are entitled to a one-year maternity leave but partly funded childcare is only available once children turn 3. As a result, mothers tend to adjust their working patterns (Khoudja & Platt, 2018; Mikolai & Kulu, 2022a). A one-year maternity leave was introduced in 2003; prior to this, 18 weeks of maternity leave and a basic allowance was available since 1975 (Moss & O'Brien, 2019). Statutory maternity pay was introduced in 1987 (Long, 2012).

In Germany (especially West Germany), family policies used to be conservative, encouraging women to provide care instead of full-time employment (Kowalewska, 2023; Misra et al., 2007). Women’s role in the labour market tended to either cease or reduce upon motherhood (Kreyenfeld, 2004) leading to low female employment rates. These policies supported families and attempted to compensate women via generous allowances and parental leave (Kowalewska, 2023; Misra et al., 2007). A 10-month means-tested parental leave was introduced in 1986. In 1992, the maximum leave duration was extended to 3 years, with 2 years of paid benefits (Geisler & Kreyenfeld, 2011). In 2007, the parental leave system has changed significantly shifting from a flat-rate benefit to an earnings-related allowance (*Elterngeld*) to promote and more effectively support work–family reconciliation and maternal employment (Spiess & Wrohlich, 2008). Additionally, from 2005 the formal childcare infrastructure expanded significantly, especially aimed at younger children (Schober & Schmitt, 2013). In 2013 and 2015, additional adjustments were made to childcare availability and parental allowances (Milewski & Brehm, 2023). Now, a year-long paid leave is offered with an additional two months if both parents take leave (Spiess & Wrohlich, 2008).

French family policy is generous and comprehensive (Pailhé & Solaz, 2013). Policies encourage women to provide care and engage in employment through the provision of high-quality public subsidised childcare, generous parental leave, and support for part-time employment (Kowalewska, 2023; Misra et al., 2007). These policies have changed from focusing on financial compensation for stay-at-home mothers to encouraging both parents to share childcare responsibilities. Flexible and inclusive measures were introduced in 2004 offering families greater autonomy in managing work and care responsibilities (Collombet, 2021). As a result, many children attend formal childcare and female labour market participation is high (Pailhé & Solaz, 2013). However, the low levels of benefits for stay-at-home mothers disproportionately affect low-income women, encouraging them to choose caregiving over employment (Kowalewska, 2023).

Most studies have not considered the impact of family policies on immigrants and descendants (Milewski & Brehm, 2023; Mussino et al., 2023). However, due to different eligibility criteria, immigrants may not be entitled to parental leave or may receive fewer benefits than others (Duvander & Koslowski, 2023). A few studies have addressed these questions showing differences between migrants and non-migrants (Andersson et al., 2006; Maes et al., 2023; Milewski & Brehm, 2023; Mussino & Duvander, 2016).

### Rights of Immigrants and Their Descendants

Access to benefits might differ between native and migrant populations from different origins. The UK has a ‘restrictive incorporation’ regime according to Sainsbury’s (2012) classification. Both UK and Commonwealth citizens used to enjoy citizenship rights including full access to social benefits (except social housing) and employment (Sainsbury, 2012). These rights were gradually removed, and by the 1980s immigrants had to be able to support themselves and their families without accessing public funds (Sainsbury, 2012). To access financial support, immigrants had to acquire permanent residence (Sainsbury, 2012). After 1981, British citizenship was no longer automatically awarded to those who were born in the country and access to social assistance was restricted based on citizenship (Sainsbury, 2012). Since the 2000s, requirements for naturalisation have become more restrictive and citizenship is awarded on a discretionary basis (Sainsbury, 2012).

Germany also has a ‘restrictive incorporation’ regime (Sainsbury, 2012). German ethnicity has been an advantage in acquiring citizenship, exemplified by the favourable legal path towards full integration for ethnic German immigrants, including the possibility of gaining dual citizenship, compared to non-ethnic Germans, who could only apply for naturalisation after a long period (15 years before 2000 and 8 years since 2000) of continuous residence (Sainsbury, 2006). Social rights are often tied to employment. Before 2005, immigrant women often faced employment restrictions leading to economic dependence even though employed immigrants were entitled to the same social rights and benefits as employed native Germans (Sainsbury, 2006). After 2005, access to social rights and employment-related benefits became universal regardless of immigration status (Kohlmeier et al., 2006).

France has an ‘inclusive incorporation’ regime (Sainsbury, 2012), where naturalisation and assimilation are the norm. The right to citizenship is based on place of birth either at birth (if one parent was born in France) or upon reaching adulthood (Sainsbury, 2012) regardless of residence. This is important, given that access to equal rights strongly depends on nationality (Nicholls, 2012). Residence in France is a requirement to gain access to certain rights (Isidro & Math, 2020). As the social insurance system is based on employment contributions, foreign workers have access to many benefits shortly after arrival (Sainsbury, 2012). Pro-natalist measures have led to a rise in allowances and benefits available to families (Pailhé et al., 2008; Thévenon, 2016).

We expect that mothers (natives, immigrants, and their descendants) in France will be the most likely to enter employment and the least likely to exit it whereas mothers in Germany will be the least likely to (re-)enter and the most likely to exit employment. Mothers’ employment (re-)entry and exit patterns in the UK are expected to be in-between those of French and German mothers (H3; country context hypothesis).

## Data

For the UK, we use Waves 1 to 9 (2009–2019) of the UK Household Longitudinal Study (UKHLS); a nationally representative panel of approximately 30,000 households (University of Essex, 2020). The UKHLS contains detailed and reliable retrospective information on the year and month of employment changes and the birth of all children since age 16. Additionally, prospective information on changes in employment status and the birth of (additional) children is collected annually. Although employment histories in the UKHLS have only been collected for a subset of individuals, previous studies have shown that the employment sample does not seem to be selective when studying immigrants’ and descendants’ partnership, fertility, and employment trajectories (Mikolai & Kulu, 2022a, 2025). The UK analytical sample consists of 12,607 native, 1922 immigrant, and 3142 descendant women.

For Germany, we use the German Socio-Economic Panel (SOEP, version 37; 1984–2020); a panel study of over 19,000 households (Wagner et al., 2007), including the migration (IAB-SOEP) and refugee (IAB-BAMF-SOEP) samples. We study West Germany, where most of the foreign-born populations live. The SOEP collects yearly retrospective information on childbirth and employment changes from age 16. As many events can happen in the same year, we assume that immigration (for immigrants) or leaving education (for natives and immigrants’ descendants) happen at the beginning of the year, followed by entering employment, exit from employment, and employment re-entry. Those who have a first child and exit employment in the same year are considered to have exited in the previous year to ensure the comparability of data across countries. As partnership histories are only available for individuals who have been observed since age 17 and who answered the biography questionnaire in wave 28 (2012) or later, incorporating partnership status in the analyses has reduced the sample size from 26,530 to 14,391. Additional analyses (not shown) revealed similar rates of employment (re-)entry and exit in the two datasets. The German analytical sample includes 10,864 native, 1397 immigrant, and 2130 descendant women.

For France, we use the Trajectories and Origins^4^ (TeO; 2008/2009) survey (Beauchemin et al., 2016), which contains information on a nationally representative sample of more than 20,000 individuals, including immigrants, immigrants’ descendants, and French natives. The survey collected retrospective information on the year and month of childbirths. Additionally, it contains retrospective yearly information on individuals’ education and employment status starting from the time of arrival for immigrants and from the time of birth for natives and descendants. We convert employment histories to a monthly format assuming that employment changes occur at the end of the year. If childbirth and employment change happen in the same month, we assume that childbirth precedes employment change. The French analytical sample includes 1842 native, 2923 immigrant, and 5365 descendant women.

Information in the three datasets is of high quality and highly comparable. These datasets provide a unique opportunity to study the lives of immigrants and descendants across countries from different origin countries. The UKHLS includes two immigrant and ethnic minority boost samples ensuring a sufficiently large sample size among individuals from the largest ethnic groups (McFall et al., 2019). The SOEP has oversampled individuals with a migration background (Jacobsen et al., 2021). Additionally, in 2013, around 2700 households were interviewed, each containing at least one person with a migration background (Brücker et al., 2014). Finally, TeO has oversampled certain origins to provide ample information on minorities who are typically underrepresented in demographic surveys (Beauchemin et al., 2016). In addition to detailed information about migrant origin, migration background, employment changes and childbirths, all datasets contain information on individuals’ socio-demographic characteristics such as birth cohort, year of migration, partnership changes, and educational level. We select individuals who were born after 1940.

## Methods

We estimate a series of piecewise constant hazard models to study transitions into first employment, out of first employment, and employment re-entry across countries and population subgroups. We use the count data approach, which is useful for harmonising cross-national data from large national longitudinal datasets when the analysis is to be executed on a pooled dataset. For each country, we prepare an occurrence-exposure table, defined by cross-classifying over a set of time intervals and variable categories (Preston, 2005). The cells of the resulting table include the number of events (*E*_*jk*_) and risk time (*R*_*jk*_, i.e. person-months) for each combination of covariate categories (*x*_*jk*_) for each time period *j* and variable category *k*. We describe the models based on Kulu et al. (2021) and Kulu et al. (2017). For each cell, the hazard or rate (${\lambda }_{jk}$) is calculated as the total number of events divided by the risk time:

$${\lambda }_{jk}= \frac{{E}_{jk}}{{R}_{jk}}$$

Let ${E}_{jk}$ denote the number of employment transitions (i.e. entry, exit, or re-entry) for group *k* in time period *j*. ${E}_{jk}$ is the realisation of a Poisson random variable with mean ${\mu }_{jk}$. The expected number of employment transitions is, thus, the product of the hazard of employment transitions and exposure:

$${\mu }_{jk}={\lambda }_{jk} x {R}_{jk}$$

In a log-linear format, this is equivalent to:

$$\text{ln}{\mu }_{jk}=\text{ln}{\lambda }_{jk}+\text{ln}{R}_{jk}$$

Then, we rearrange the equation to investigate the hazard of employment transitions:

$$\text{ln}\left(\frac{{\mu }_{jk}}{{R}_{jk}}\right)=\text{ln}{\lambda }_{jk}$$

Finally, the log-linear model for the hazard of employment (re-)entry and exit, which also includes covariates, is specified as:

$$\text{ln}{\mu }_{jk}={\alpha }_{j}+{\beta x}_{k}$$

where ${\alpha }_{j}$ is the baseline hazard, i.e. the hazard of employment (re-)entry or exit by the relevant duration variables (see below); ${x}_{k}$ is a vector of the covariates and $\beta$ is a vector of parameters to measure the effect of the covariates.

We pool the occurrence-exposure datasets for three countries and estimate a series of Poisson regressions on these data. This approach is ideal for cross-national comparisons as it allows us to compare different migrant/origin groups with each other within and across countries using only one reference group as the benchmark (see, for example, Hannemann et al., 2020; Kulu et al., 2017, 2024). Estimating a Poisson regression on occurrence-exposure data is equivalent to estimating piecewise constant hazard models with categorical variables (Holford, 1980; Laird & Olivier, 1981).

**Fig. 1:**
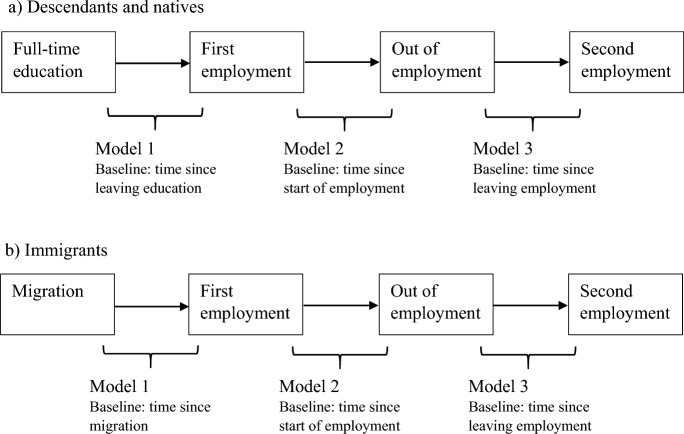
Modelling strategy. *Note* In the models for descendants and natives, the reference group is native women in the UK. In the models for immigrants, the reference group is European/Western immigrants in the UK. For immigrants, entry into first employment refers to entering employment for the first time in the destination country following migration

We conduct the analyses in three steps separately for immigrants as well as descendants and natives (Fig. 1). Model 1 estimates the risk of entering first employment among immigrants and descendants. Among immigrants, this means entering first employment in the destination country. The baseline differs between immigrants and descendants. Among the descendants and natives, those who left full-time education are at risk of entering first employment; hence, the baseline is time since leaving education (0–1 year, 1–3 years, 3–5 years, and 5 + years). Among immigrants, the baseline is time since migration (0–1 year, 1–3 years, 3–5 years, and 5 + years) as they enter the risk set after arriving in the destination countries. For immigrants whose employment started in the same year and month as when they have arrived in the destination countries, we have imputed a one-month waiting time. Among descendants, if information is not available on the age at leaving education, we imputed the average age of leaving education by level of education.^5^

Model 2 estimates the risk of exiting first employment among those who entered first employment. The baseline is time since start of first employment for immigrants, descendants, and natives. Finally, Model 3 estimates the risk of re-entering employment among those who exited first employment; the baseline is time since leaving first employment among immigrants, descendants, and natives. Model 3 is only estimated for the UK and Germany as information on employment re-entry is not available for France. For the definition of being employed or not, see the Variables section.

In all models, we first examine the role of migrant origin (in case of immigrants) or migration background (in case of the descendants) and parity separately on the risk of the examined transitions whilst controlling for important compositional variables. Second, we include interaction terms between migration origin/background and parity to understand whether mothers with different migration origin/background have different patterns of employment entry and exit than childless women from the same group. As we estimate separate models for immigrants as well as descendants and natives, the estimates from the models are not directly comparable. However, we can indirectly compare patterns and trends in the levels of immigrants’ and descendants’ employment (re-)entry and exit rates. When comparing across generations, we calibrate our indirect comparisons by comparing the experiences of European immigrants and descendants across countries. Individuals are observed from age 15 or age at arrival (among immigrants)/age at leaving education (among natives and descendants), whichever was first until age 50 or the end of the observation.

## Variables

Employment status is key to the construction of the dependent variables (entry into first employment, employment exit, employment re-entry). Individuals can either be employed for the first or second time (i.e. in paid part- or full-time employment, salaried, self-employed, military service, or on maternity leave) or not employed (i.e. unemployed, looking for work, looking after family or home, homemaker, student, long-term sick or disabled, on a government training scheme, something else, retired, or not in the labour force).

Migration origin/background is the key independent variable; it is determined using information on individuals’ own and their parents’ country of birth. Natives are individuals who were born in the destination countries to two parents also born in the destination countries. Immigrants were born outside the destination countries. Descendants were born in the destination countries, but at least one of their parents was born abroad. In this study, the 1.5 generation (i.e. those who migrated before age 15) is included with the second generation as those who migrated as children are more similar to the descendants than to immigrants who arrived at later ages regarding their exposure to the destination country’s norms and institutions. Indeed, additional analyses (not shown) revealed that the patterns of the 1.5 and second generation are very similar. To derive this variable, in the UK we combine information on individuals’ and their mothers’ country of birth (or ethnicity if country of birth is missing and information on father’s country of birth or ethnicity if the mother’s information is missing or the mother is UK-born). In Germany and France, we use information on respondents’ own and their parents’ country of birth and citizenship. The resulting groups are immigrants and descendants from European and Western countries, India, Pakistan, Bangladesh, the Caribbean, and African countries in the UK; North Africa, sub-Saharan Africa, South-East Asia, Turkey, Southern Europe, and other European countries in France; and Poland, Russia/Kazakhstan, Southern Europe, and Turkey in Germany.^6^ These are the main groups of immigrants who also already have descendants in the destination countries and where we have sufficient numbers of individuals to conduct detailed analyses. In the analyses of natives and the descendants of immigrants, the reference category is natives in the UK. This allows us to compare the patterns of the descendants of immigrants to those of the natives and to help us better understand the role of adaptation and socialisation. In the analysis of immigrants, the reference category is European and Western immigrants in the UK because they are the most similar to UK natives aiding the indirect comparison across the two sets of models. Choosing the reference groups to be from the UK is a technical consideration; we could have chosen native French or native German women, and the results would not change because the risks of each group are calculated relative to the reference group.

Next to the relevant duration variables, we also control for relevant age variables such as age at leaving education (Model 1 for descendants categorised as < 20 or 20 +), age at migration (Model 1 for immigrants grouped as 16–19, 20–24, or 25 +), age at starting first employment (Model 2 for all individuals categorised as < 20, 20–24, 25–29, 30 +), and age at leaving first employment (Model 3 for all individuals grouped as < 25, 25–29, 30–34, 35 +). The analyses are also adjusted for the most important compositional variables which we have comparable data for and that have been shown to be important for immigrants’ and descendants’ employment transitions. These include level of education (low, medium, high), birth cohort (for descendants and natives categorised as 1940–1949, 1950–1959, 1960–1969, 1970–1979, 1980–1989, 1990 +) or migration cohort (for immigrants grouped as 1956–1989, 1990–1999; 2000 +), and partnership status (single, cohabiting, married, separated). Parity is categorised as having no children vs having one or more children. Tables 1, 2, 3 and 4 in Appendix show the number of events and person-months by covariate categories.

## Results

### First Employment Entry

**Fig. 2:**
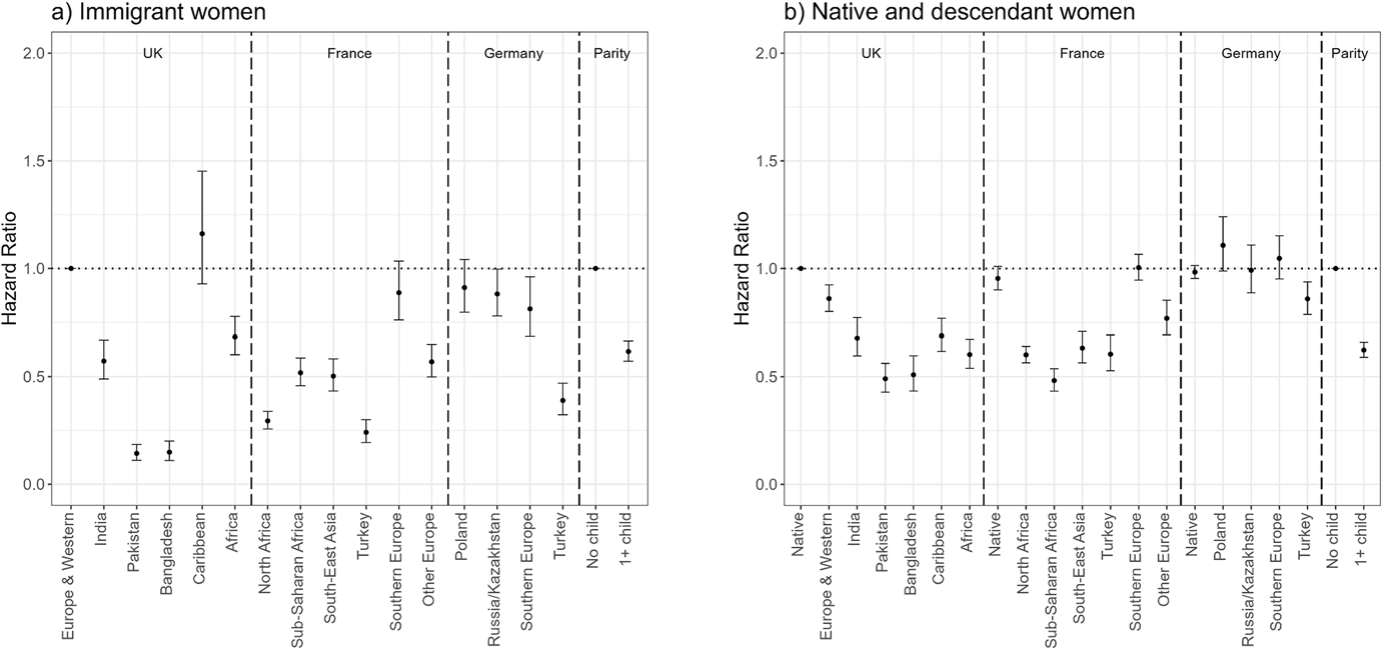
Hazard ratios of first employment entry among immigrant women (panel a) and female natives and descendants (panel b) by migrant origin and parity (main effects). *Source* Authors’ calculations using data from the UK Household Longitudinal Study (UKHLS) for the UK, Trajectories and Origins (TeO) for France, and the German Socio-Economic Panel (SOEP) for Germany. *Note* Whiskers indicate 95% confidence intervals compared to the reference category (European and Western immigrants in the UK for panel a) and native women in the UK for panel b)). Full regression results are shown in Appendix Table 5 (immigrants) and Table 6 (natives and descendants). Results for immigrants (panel a) and natives and descendants (panel b) are from separate models and hence are not directly comparable

Figure 2 shows the hazard ratios of first employment entry (Model 1) among immigrant women (panel a) and female natives and descendants (panel b). For immigrants, the reference category is the risk of European and Western immigrants in the UK to enter first employment, whereas for the descendants, it is the risk of native women in the UK to do so. The first employment entry risks of all groups are shown in relation to these reference groups, but by assessing whether confidence intervals overlap, we can compare relative risks of different groups both within and across countries. In the UK, all groups (except Caribbeans) have significantly lower rates of entry into first employment than European/Western immigrants. The lowest rates of first employment entry are among Pakistani and Bangladeshi immigrants, whereas these rates are the highest among European/Western and Caribbean immigrants. Immigrants’ first entry risks from India and Africa are in-between these two groups. In France, Southern European immigrants have the highest risks to enter first employment after arrival (not statistically different from the risks of European/Western immigrants in the UK), whereas those from Turkey and North Africa have the lowest risks. The entry risks of other migrant groups are in-between the risks of these two groups. Finally, in Germany, Turkish immigrants have the lowest first employment entry risks after arrival; the other groups are all more likely to enter first employment. Polish and Russian/Kazakh immigrants in Germany are as likely as European/Western immigrants in the UK to enter first employment, whereas Southern European immigrants in Germany are somewhat less likely to do so. Regarding the role of childbearing, immigrant women who have children have a 40% lower likelihood of entering first employment following migration than those who are childless.

Among native women and second-generation origin groups, although the magnitude of the differences between the entry risks of native women and descendants is smaller than the differences were between European/Western and other immigrants, again, European/Western descendants had higher risks of first employment than other groups. In the UK, all descendant groups (particularly Pakistanis and Bangladeshis) have lower risks of first employment entry than native women. In France, Southern European descendants have similar entry risks to native French women whereas North African, sub-Saharan African, South-East Asian, Turkish, and other European descendants are less likely to enter first employment than native women. We see far fewer differences between the first employment entry risks of natives and different groups of descendants in Germany. Those with Turkish origin have lower risks of first employment entry than native German women, whereas all other groups have comparable risks to native women. Regarding the role of parity, mothers are less likely to enter first employment than childless women among native women and the second generation.

**Fig. 3:**
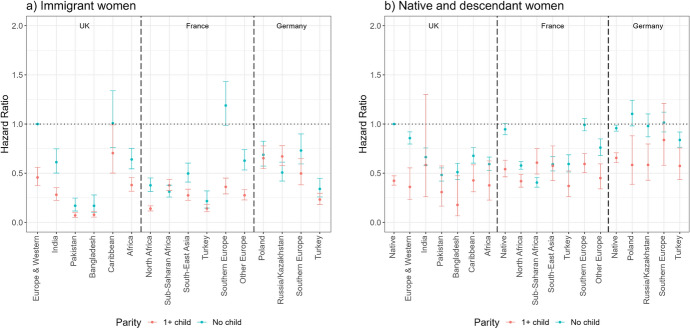
Hazard ratios of first employment entry among immigrant women (panel a) and female natives and descendants (panel b) by migrant origin and parity (interaction effects). *Source* Authors’ calculations using data from the UK Household Longitudinal Study (UKHLS) for the UK, Trajectories and Origins (TEO) for France, and the German Socio-Economic Panel (SOEP) for Germany. *Note* Whiskers indicate 95% confidence intervals compared to the reference category (childless European and Western immigrants in the UK for panel a) and childless native women in the UK for panel b)). Full regression results are shown in Appendix Table 7 (immigrants) and Table 8 (natives and descendants). Results for immigrants (panel a) and natives and descendants (panel b) are from separate models and hence are not directly comparable

The interaction models reveal that the role of motherhood for entering first employment is largely similar across all migrant groups and countries. Overall, immigrant mothers are less likely to enter first employment than childless immigrant women (Fig. 3, panel a). The differences between the entry risks of childless women and mothers are most pronounced (and statistically significant) among immigrants from Europe/Western countries, India, and Africa in the UK and among immigrants from Europe, North Africa, and South-East Asia in France. Although the differences between different groups of immigrant mothers’ and childless immigrant women’s first employment entry risks are not significant in Germany, the patterns are similar (except among Russian/Kazakh immigrants) to what is reported above. The overall patterns are similar among native women and the female descendants of immigrants across countries: childless women are more likely to enter employment than mothers. The differences are significant among native women and European/Western descendants in the UK, native women and European and North African descendants in France, and native women and Polish and Russian/Kazakh descendants in Germany. A notable difference is sub-Saharan African descendants in France where mothers have a higher risk of entering first employment than childless women. The reasons for this are unclear and warrant further research.

### Employment Exit

**Fig. 4:**
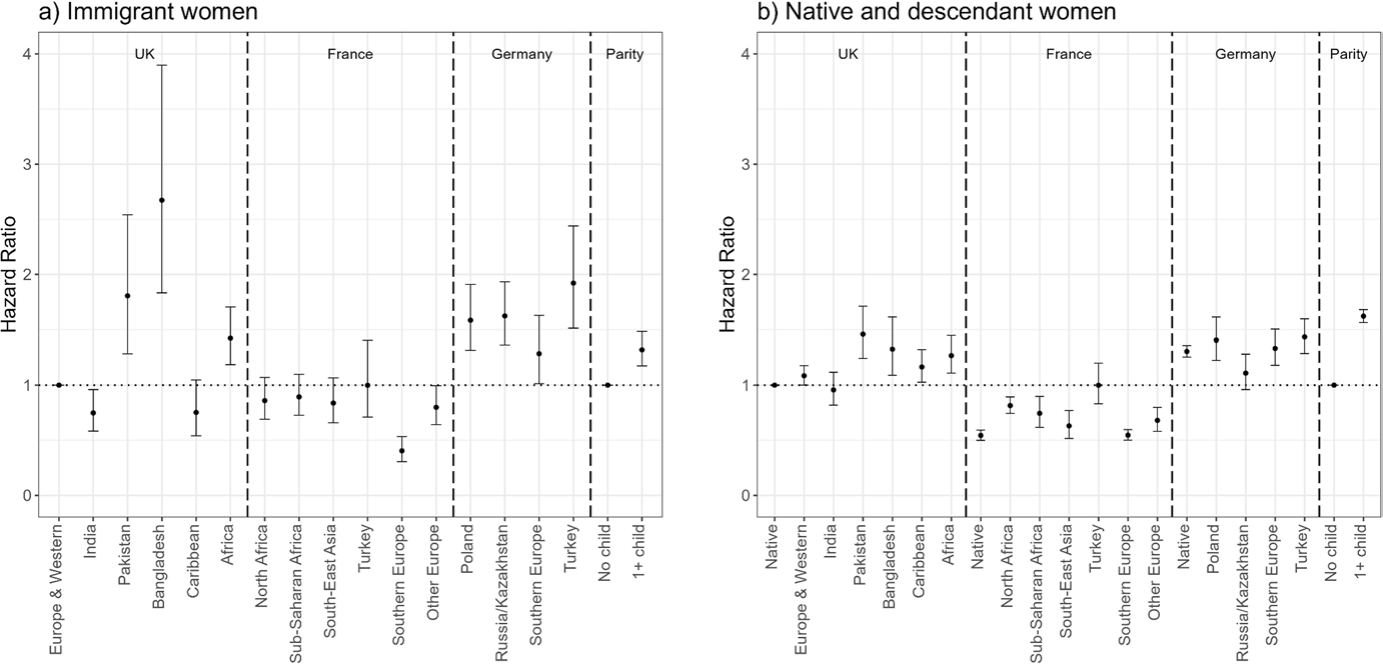
Hazard ratios of employment exit among immigrant women (panel a) and female natives and descendants (panel b) by migrant origin and parity (main effects). *Source* Authors’ calculations using data from the UK Household Longitudinal Study (UKHLS) for the UK, Trajectories and Origins (TeO) for France, and the German Socio-Economic Panel (SOEP) for Germany. *Note* Whiskers indicate 95% confidence intervals compared to the reference category (European and Western immigrants in the UK for panel a) and native women in the UK for panel b)). Full regression results are shown in Appendix Table 5 (immigrants) and Table 6 (natives and descendants). Results for immigrants (panel a) and natives and descendants (panel b) are from separate models and hence are not directly comparable

Figure 4 shows the hazard ratios of exiting first employment (Model 2) among immigrants (panel a) and native women and the female descendants of immigrants (panel b). The reference categories are the risks of European/Western immigrants in the UK for immigrants, and the risks of native women in the UK for natives and descendants. Again, all groups’ risks of exiting employment are calculated in relation to the reference categories, but by assessing whether confidence intervals overlap, we can compare relative risks of different groups both within and across countries. First, some groups of immigrants are more likely to exit first employment than European immigrants across countries. For example, in the UK, Pakistani, Bangladeshi, and African immigrants have higher exit risks than European/Western immigrants. At the same time, Caribbean immigrants have comparable risks to European/Western immigrants whereas Indian immigrants have somewhat lower risks. In France, all immigrant groups have a significantly higher likelihood of exiting first employment than Southern European immigrants. In Germany, we find smaller and fewer significant differences between different immigrant groups; nonetheless, Turkish immigrants tend to have the highest and Southern Europeans the lowest likelihood of exiting first employment. Regarding the role of motherhood, immigrant mothers have higher risks of exiting first employment than childless women.

Overall, we find smaller differences in the likelihood of employment exit among natives and the descendants of immigrants than among immigrants. In the UK, Pakistani, Bangladeshi, and African second-generation women stand out as having a higher likelihood than native women to exit first employment. In France, North African, sub-Saharan African, and European descendants are more likely to exit employment than French natives, whereas in Germany, second-generation Russian and Kazakh women are somewhat less likely to exit employment than native Germans (differences are not significant). The lower overall exit risks among French compared to British and German native women are in line with previous studies, showing that employment rates among women are lowest in France (Algan et al., 2010). Additionally, native and second-generation mothers are more likely to exit employment than childless women.

**Fig. 5:**
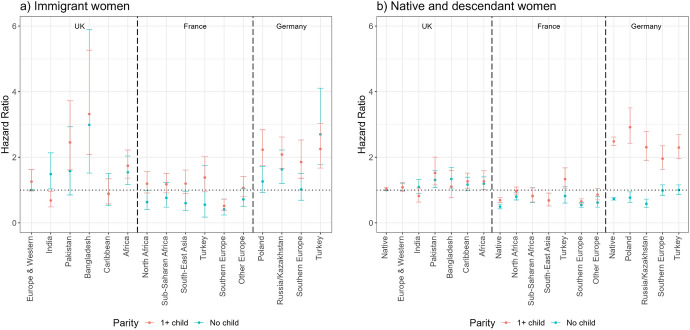
Hazard ratios of employment exit among immigrant women (panel a) and female natives and descendants (panel b) by migrant origin and parity (interaction effects). *Source* Authors’ calculations using data from the UK Household Longitudinal Study (UKHLS) for the UK, Trajectories and Origins (TEO) for France, and the German Socio-Economic Panel (SOEP) for Germany. *Note* Whiskers indicate 95% confidence intervals compared to the reference category (childless European and Western immigrants in the UK for panel a) and childless native women in the UK for panel b)). Full regression results are shown in Appendix Table 7 (immigrants) and Table 8 (natives and descendants). Results for immigrants (panel a) and natives and descendants (panel b) are from separate models and hence are not directly comparable. Large confidence intervals are related to small number of events

The interaction models (Fig. 5) show that the patterns by parity largely hold among all immigrant and descendant groups. We note the large confidence intervals and often not significant differences between immigrant mothers and childless women. We find the most remarkable differences in Germany; German native women as well as all groups of descendants of immigrants are considerably more likely to exit employment if they have children than if they are childless.

### Employment Re-entry

**Fig. 6:**
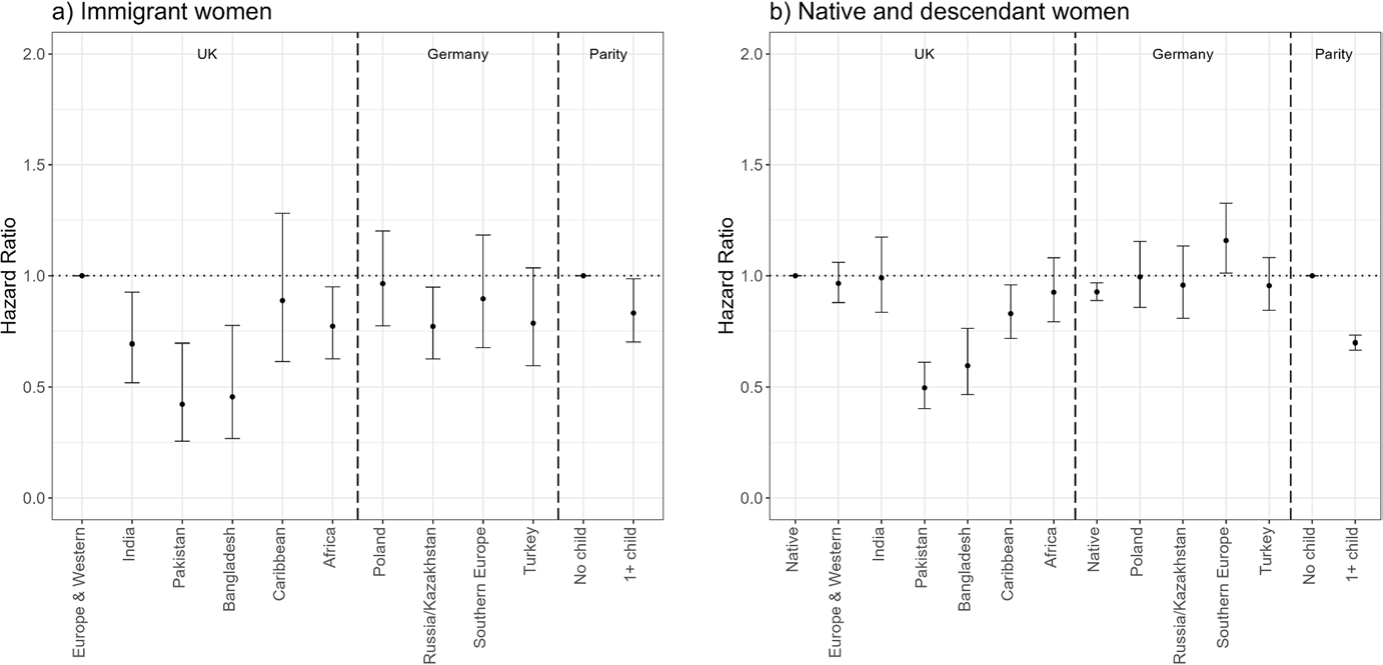
Hazard ratios of employment re-entry among immigrant women (panel a) and female natives and descendants (panel b) by migrant origin and parity (main effects). *Source* Authors’ calculations using data from the UK Household Longitudinal Study (UKHLS) for the UK, and the German Socio-Economic Panel (SOEP) for Germany. *Note* Whiskers indicate 95% confidence intervals compared to the reference category (European and Western immigrants in the UK for panel a) and native women in the UK for panel b)). Full regression results are shown in Appendix Table 5 (immigrants) and Table 6 (natives and descendants). Results for immigrants (panel a) and natives and descendants (panel b) are from separate models and hence are not directly comparable

Figure 6 shows the results of Model 3 (employment re-entry) for the UK and Germany.^7^ For immigrants (panel a), the reference group is the re-entry risks of European/Western immigrants in the UK, whereas for the descendants (panel b), it is the re-entry risks of native women in the UK. All immigrant and descendant groups’ risks of employment re-entry are calculated in relation to the reference categories, but by assessing whether confidence intervals overlap, we can compare relative risks of different groups both within and across countries. In the UK, Indian, Pakistani, Bangladeshi, and African immigrants are less likely to re-enter employment than European/Western immigrants. Caribbean immigrants have comparable employment re-entry risks to European/Western immigrants. There are no significant differences between the re-entry risks of immigrant women from different origin countries in Germany. Regarding the role of motherhood, childless immigrant women are somewhat more likely to re-enter employment than mothers.

Among the descendants, Pakistani, Bangladeshi, and Caribbean descendants in the UK are less likely to re-enter employment than native women, whereas in Germany, Southern European descendants have somewhat higher likelihood to re-enter employment than native women. Childless native women and female descendants are more likely to re-enter employment than mothers.

**Fig. 7:**
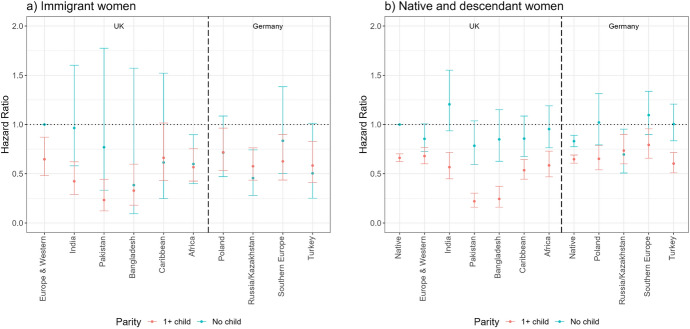
Hazard ratios of employment re-entry among immigrant women (panel a) and female natives and descendants (panel b) by migrant origin and parity (interaction effects). *Source* Authors’ calculations using data from the UK Household Longitudinal Study (UKHLS) for the UK, and the German Socio-Economic Panel (SOEP) for Germany. *Note* Whiskers indicate 95% confidence intervals compared to the reference category (childless European and Western immigrants in the UK for panel a) and childless native women in the UK for panel b)). Full regression results are shown in Appendix Table 7 (immigrants) and Table 8 (natives and descendants). Results for immigrants (panel a) and natives and descendants (panel b) are from separate models and hence are not directly comparable

Figure 7 shows the results of the interaction models for employment re-entry. The overall pattern of childless women being more likely to re-enter employment than mothers holds for immigrant women (panel a) although due to small numbers of events among childless women, it is difficult to draw firm conclusions. Among native women and descendants (panel b), we find significant evidence for this relationship among most origin groups. The differences in re-entry risks between mothers and childless women are particularly striking among native women, and Indian, Pakistani, and Bangladeshi descendants, but they are also significant among Caribbean and African descendants in the UK. In Germany, the differences are significant between mothers and childless women among natives as well as among Polish and Turkish descendants.

## Conclusions and Discussion

We studied the interrelationship between employment trajectories and childbearing among immigrant, descendant, and native women in the UK, France, and Germany. We found that employment changes and childbearing are interrelated not only among native women but also among immigrants and their descendants. Overall, across countries, mothers are less likely to enter employment than childless women, and, at the same time, they are more likely to exit and less likely to re-enter the labour market. However, we also detected significant heterogeneity between migrant origin groups, generations, and destination countries. These differences are more striking than the differences between the employment patterns of mothers and childless women.

First, we expected that there would be larger differences between immigrant mothers’ and childless immigrant women’s employment (re-)entry and exit levels among non-European than among European immigrant groups (H1a). Although in line with previous studies (Kil et al., 2018; Mikolai & Kulu, 2025; Vidal-Coso, 2019) we found that immigrant mothers were overall less likely to (re-)enter and more likely to exit employment than childless immigrant women, we did not find support for this expectation. On the contrary, the largest difference between childless immigrant women’s and mother’s employment entry rates were among European women. Regarding employment exit and re-entry, we found few significant differences between mothers and childless immigrant women.

These findings indicate that differences between the experiences of mothers and childless immigrant women primarily stem from *not* entering the labour market in the first place rather than from their propensity to exit and/or re-enter employment. We speculate that immigrant women who enter employment are selected and hence more resilient against challenges that lead to labour market exit. Particularly, certain groups of immigrant women (e.g. Pakistani and Bangladeshi women in the UK or Turkish women in Germany and France) tend not to enter the labour market. This may be due to challenges that non-European immigrant women face to enter the labour market such as a lack of recognised qualifications, language proficiency, or discrimination. Another explanation might be attitudes or preferences towards a traditional division of labour among these groups. Finally, for some groups (e.g. Pakistani and Bangladeshi in the UK), marriage prior to childbirth rather than childbearing may lead to reduced labour market participation (Holdsworth & Dale, 1994; Khoudja & Platt, 2018). We conclude that European immigrant women’s employment entry is influenced by childbearing to a larger extent than non-European women’s because they are more likely to be employed in the first place. Among immigrant women who are unlikely to enter the labour market, rates are low regardless of whether they have children or not. Thus, we found the largest differences in employment (re-)entry and exit risks between different origin groups and not between mothers and childless women.

Second, we expected that the influence of childbearing on the employment of second-generation women would be more similar to those of native women than immigrant women from the same origin (H2) and that this would especially be the case among European descendant groups (H1b). We found that native women and European descendants in the UK and France were significantly less likely to enter employment if they had children than if they were childless. This provides partial support for our expectation (H2) but only among European descendants (partly confirming H1b). However, we did not find such differences among the other descendant groups. This indicates that similarly to what we found among immigrants, the fact that some second-generation groups are less likely to enter employment in the first place is what leads to differences in employment trajectories and not childbearing. Hence, even among the second generation, among some groups, there may be differences in gendered norms and preferences regarding employment and childbearing, or discrimination or structural constraints may lead to some second-generation groups being less likely to enter employment in the first place. Taken together, these results indicate that heterogeneity and labour market disadvantage persist across migrant generations, providing evidence for uneven assimilation trajectories among different (especially non-European) origin groups. These results challenge the assimilation hypothesis and provide partial support for the minority group status or minority subculture hypotheses for the descendants of non-European immigrants.

At the same time, results of employment exit and re-entry provide partial evidence for our expectation that native and second-generation women would have similar patterns (H2). Childless women and mothers had similar rates of employment exit in the UK and France. Furthermore, all groups of descendants (except European/Western) and native women in the UK had similar patterns of employment re-entry, and this was also the case in Germany for native women and Turkish descendants. This suggests that among the second generation, it is also primarily entry into first employment where the patterns are most different compared to native women’s experiences. This is in line with Maes et al. (2021), showing that Belgian and second-generation mothers’ lower employment were explained by lower pre-birth labour market attachment. At the same time, the findings for employment exit and re-entry challenge the origin hypothesis among the descendants (H1b), expecting that the patterns of European immigrants would be most similar to those of the natives.

Finally, we expected that cross-national differences in work–family reconciliation and immigration policies meant that (native, immigrant, and second generation) mothers in France would be the most likely to enter and the least likely to exit employment, whereas they would be most likely to exit and least likely to enter in Germany with the UK taking an intermediate position (H3). We found support for this expectation but only regarding the propensity of employment exit among native women and the second generation. Exit risks were the smallest among French and the largest among German native and second-generation mothers. In France, both immigration and work–family policies are inclusive regarding women’s ability to combine paid work and childcare and the descendants’ ability to access benefits. On the contrary, German family policy encourages women to exit the labour market and focus on childcare whilst also restricting access to benefits among immigrant populations. These findings highlight the importance of work–family reconciliation policies and inclusive immigration policies allowing second-generation immigrants to access all benefits and support available to natives. The results highlight that such policies are most important for mitigating the consequences of childbearing for exiting employment.

This study has some limitations. First, next to employment status, working hours, type of contract, or the availability of flexible working may be important. The role of these characteristics and their interplay with motherhood might differ between immigrants and descendants as well as between different origin groups across countries. Similarly, although the analyses were adjusted for basic socio-demographic factors, other factors such as religiosity or language skills may explain differences in the experiences of immigrants and their descendants. Additionally, among immigrants, the reason for migration is likely to be an important determinant of their employment trajectories. Unfortunately, comparable information on these variables was not available in all datasets. Second, although all efforts were made to harmonise data from the three countries, some differences present challenges. Whilst the SOEP and the UKHLS have similar designs, TeO is cross-sectional. Although it contains retrospective biographical information, it did not collect information on full employment histories, and hence, data are not available on repeated employment entries. TeO was collected in 2008/2009 whilst the other two data sources follow individuals up to 2019/2020. Whilst this may lead to some issues related to different labour market and economic conditions across different time periods and countries, TeO is the comparable data that was available for France when the analysis was completed. Some questions remain unanswered. For example, the family of origin may play an important role in explaining women’s strategies to reconcile work and family. Future research could examine whether women whose mothers worked are more likely to be in the labour force and work more hours themselves following childbearing. Similarly, the origin of the partner is likely to influence women’s propensity to enter and exit the labour market following childbirth. We could also not investigate the role of the number of children and age of the youngest child in women’s propensity to (re-)enter and exit the labour market and how this interacts with migrant generation, origin, and host country context due to data limitations. Finally, we could not empirically assess whether and how changes in the policy context may modify the link between motherhood and employment. We leave these important questions for future research.

Methodological challenges to be addressed by future studies include that the models for immigrants and descendants (and natives) have different baselines, implying that in later transitions immigrants and descendants are compared at different points in their life course (e.g. differences between the nature of exit from a first post-migration job vs exit from a first post-education job) or that there may be potential selection into the study population for analysing employment re-entry. This set-up also makes it challenging to formally test the hypothesis related to change across generations. Similarly, exploring how to incorporate sample weights into the analysis when using aggregated occurrence-exposure data remains for future research.

Taken together, we have shown that overall mothers are less likely to (re)-enter and more likely to exit employment among native women as well as immigrants and descendants. However, the largest differences were not between mothers and childless women but between different origin groups, migrant generations, and destination countries. Labour market disadvantage among all mothers stem from low levels of labour market activity prior to childbearing as well as a larger propensity to exit the labour market following childbearing. Our study highlights the importance of work–family reconciliation and immigration policies for reducing labour market disadvantage among mothers overall and particularly among immigrant and second-generation mothers. To do so, policies need to enable women to enter the labour market in the first place and to remain economically active following childbirth.

## Data Availability

All datasets used in this analysis are available to download after registration with the relevant organisations. The UK Household Longitudinal Study is hosted and managed by the UK Data Service, the German Socio-Economic Panel is available from the German Institute for Economic Research, and the Trajectories and Origins survey (Trajectoires et Origines (TeO 1)) is produced by the National Institute of Statistics and Economic Studies (INSEE) and the French Institute for Demographic Studies (INED) and distributed by the National Archive of Data from Official Statistics (ADISP-CMH).

## Appendix

Note 1. Information on imputing the average age at leaving education.

Imputing the average age of leaving education is necessary in the UK (the mean ages of leaving education are 16, 18, and 21 for low, medium, and highly educated women respectively) and Germany (the mean ages are 17.8, 18.8, and 21.2 for low, medium, and highly educated women respectively). In France, there is no missing information on the age of leaving education. However, there is a lot more variability in the age at which individuals with the same level of education leave education. As we assume a common baseline among native women and the descendants of immigrants across the three countries, these differences in the age pattern of leaving education in France compared to the other two countries have an impact on the results leading to depressed rates of entry into first employment not because of lower levels of entry but because of a later timing of first employment entry for some individuals. To solve this issue, we have replaced the age of leaving education with the mean age of leaving education by level of education in France (the mean ages are 18, 20, and 24 for low, medium, and highly educated individuals, respectively). For the small proportion of the sample who leaves education and enters first employment before age 18, we retain the original information so we can keep them in the sample.

See Tables 1, 2, 3, 4, 5, 6, 7, 8 and 9.

**Table 1:** Number of events and person-months among immigrant women in the UK, France, and Germany by categories of covariates included in the analyses for first employment entry and re-entry

		First employment entry						Employment re-entry			
		UK		France		Germany		UK		Germany	
		Events	Person months	Events	Person months	Events	Person months	Events	Person months	Events	Person months
Time since migration	0–1 year	761	15,140	631	27,580	757	12,975				
	1–3 years	207	23,463	373	45,700	74	14,746				
	3–5 years	174	17,625	296	34,314	97	11,402				
	5 + years	286	68,072	634	119,509	272	32,483				
Age at migration	15–19	244	33,827	295	41,873	221	25,912				
	20–24	472	46,843	675	85,693	260	20,654				
	25 +	712	43,630	964	99,536	719	25,040				
Time since employment exit	0–1 year							226	6672	304	8503
	1–3 years							137	7703	142	8120
	3–5 years							59	4684	64	4781
	5 + years							82	12,055	92	8596
Age at employment exit	< 25							108	7208	10	274
	25–29							136	9163	76	5003
	30–34							110	7263	159	9425
	35 +							150	7480	357	15,298
Migrant origin	1G Europe & Western	583	18,773					195	8964		
	1G India	230	16,964					63	4853		
	1G Pakistan	68	31,738					17	2056		
	1G Bangladesh	48	25,714					15	1619		
	1G Caribbean	92	3663					35	2160		
	1G Africa	407	27,449					179	11,462		
	1G North Africa			375	79,106						
	1G Sub-Saharan Africa			513	51,168						
	1G South-East Asia			277	24,897						
	1G Turkey			100	28,928						
	1G Southern Europe			273	17,373						
	1G Other Europe			396	25,630						
	1G Poland					351	13,804			181	7155
	1G Russia/Kazakhstan					521	24,224			270	14,354
	1G Southern Europe					181	7677			74	3043
	1G Turkey					147	25,901			77	5447
Migration cohort	1956–1989	326	43,875	913	135,794	109	10,713	125	13,136	61	3986
	1990–1999	297	37,263	588	55,700	465	35,223	135	8470	239	13,291
	2000 +	805	43,162	433	35,608	626	25,670	244	9508	302	12,723
Parity	No child	906	34,900	859	50,584	475	21,895	148	4707	81	2938
	One or more children	522	89,399	1075	176,518	725	49,711	356	26,406	521	27,062
Partnership status	Single	570	25,728	429	31,973	252	15,176	77	3248	37	1984
	Cohabiting	136	3,921	189	12,335	140	4032	51	1782	54	2347
	Married	600	85,858	1150	167,585	725	49,354	317	22,484	458	23,428
	Separated	122	8,792	166	15,209	83	3044	59	3600	53	2241
Level of education	Low	458	79,318	753	136,884	435	39,746	153	14,844	205	11,628
	Medium	347	22,328	651	67,104	451	21,416	95	4941	236	11,454
	High	623	22,654	530	23,114	314	10,444	256	11,328	161	6917
Total		1428	124,299	1934	227,102	1200	71,606	504	31,113	602	30,000

*Source* Authors’ calculations using data from the UK Household Longitudinal Study (UKHLS) for the UK, Trajectories and Origins (TeO) for France, and the German Socio-Economic Panel (SOEP) for Germany

**Table 2:** Number of events and person-months among immigrant women in the UK, France, and Germany by categories of covariates included in the analyses for employment exit

		First employment exit					
		UK		France		Germany	
		Events	Person months	Events	Person months	Events	Person months
Time since start of employment	0–1 year	200	15,248	45	21,862	159	12,477
	1–3 years	162	23,489	174	37,344	233	19,238
	3–5 years	90	18,168	128	30,915	152	13,668
	5 + years	227	74,962	299	131,263	226	37,153
Age at start of employment	< 20	76	18,780	46	21,699	32	3856
	20–24	208	43,650	158	64,554	213	20,593
	25–30	203	35,462	203	57,796	225	26,054
	30 +	192	33,975	239	77,335	300	32,033
Migrant origin	1G Europe & Western	250	52,690				
	1G India	86	22,991				
	1G Pakistan	39	4263				
	1G Bangladesh	31	1920				
	1G Caribbean	43	16,242				
	1G Africa	230	33,763				
	1G North Africa			137	40,846		
	1G Sub-Saharan Africa			169	44,374		
	1G South-East Asia			102	37,382		
	1G Turkey			41	9895		
	1G Southern Europe			70	47,970		
	1G Other Europe			127	40,917		
	1G Poland					225	22,989
	1G Russia/Kazakhstan					343	35,668
	1G Southern Europe					98	13,876
	1G Turkey					104	10,004
Migration cohort	1956–1989	167	59,204	336	154,939	72	12,713
	1990–1999	174	30,816	203	51,058	300	38,374
	2000 +	338	41,846	107	15,387	398	31,449
Parity	No child	289	51,090	118	50,247	178	21,329
	One or more children	390	80,776	528	171,137	592	61,207
Partnership status	Single	131	24,630	54	25,311	78	9522
	Cohabiting	70	11,600	62	16,214	79	8600
	Married	409	81,990	441	152,277	557	57,928
	Separated	69	13,646	89	27,582	56	6486
Level of education	Low	245	43,563	282	101,244	278	29,443
	Medium	137	24,546	207	70,184	294	31,832
	High	297	63,757	157	49,955	198	21,262
Total		679	131,866	646	221,383	770	82,536

*Source* Authors’ calculations using data from the UK Household Longitudinal Study (UKHLS) for the UK, Trajectories and Origins (TeO) for France, and the German Socio-Economic Panel (SOEP) for Germany

**Table 3:** Number of events and person-months among the female descendants of immigrants and native women in the UK, France, and Germany by categories of covariates included in the analyses for first employment entry and re-entry

		First employment entry						Employment re-entry			
		UK		France		Germany		UK		Germany	
		Events	Person months	Events	Person months	Events	Person months	Events	Person months	Events	Person months
Time since leaving education	0–1 year	8279	104,693	2721	47,894	9881	102,667				
	1–3 years	3268	69,210	1505	54,845	243	35,585				
	3–5 years	469	29,911	605	30,383	195	27,289				
	5 + years	605	134,539	705	97,794	707	136,722				
Age at leaving education	< 20	10,229	290,867	1783	82,090	6859	215,823				
	20 +	2392	47,486	3753	148,827	4167	86,440				
Time since employment exit	0–1 year							2370	89,660	2938	82,045
	1–3 years							1654	124,297	1363	79,435
	3–5 years							856	91,458	607	50,217
	5 + years							2445	297,477	1166	139,056
Age at employment exit	< 25							1511	133,879	295	10,349
	25–29							2604	226,788	1983	112,353
	30–34							1631	135,697	1728	119,625
	35 +							1579	106,528	2068	108,426
Migration background	Natives	10,547	257,498	1588	48,510	9394	263,106	6208	516,442	5250	312,446
	2G Europe & Western	817	22,981					470	36,479		
	2G India	234	8107					136	9241		
	2G Pakistan	216	13,326					90	11,318		
	2G Bangladesh	155	8450					65	5744		
	2G Caribbean	330	14,840					191	14,148		
	2G Africa	322	13,152					165	9521		
	2G North Africa			1224	75,378						
	2G Sub-Saharan Africa			360	23,246						
	2G South-East Asia			320	12,715						
	2G Turkey			220	12,361						
	2G Southern Europe			1438	41,435						
	2G Other Europe			386	17,272						
	2G Poland					311	5822			183	10,209
	2G Russia/Kazakhstan					331	8117			144	5716
	2G Southern Europe					447	8231			223	8164
	2G Turkey					543	16,987			274	14,217
Birth cohort	1940–49	2137	57,347	91	3894	550	12,865	1392	178,561	270	48,139
	1950–59	2394	72,634	710	35,533	880	14,114	1502	147,243	459	56,371
	1960–69	2631	73,097	1637	75,770	2515	99,005	1718	133,319	1648	109,239
	1970–79	2287	58,252	1929	72,768	2894	100,368	1341	89,654	1980	88,557
	1980–89	1781	37,414	1157	42,550	2362	49,272	910	42,068	1243	37,867
	1990 +	1391	39,610	12	402	1825	26,639	462	12,048	474	10,580
Parity	No child	12,152	242,852	4817	134,975	9925	174,739	2,441	95,592	1900	55,306
	One or more children	469	95,500	719	95,941	1101	127,524	4,884	507,299	4174	295,446
Partnership status	Single	11,237	212,494	3530	102,885	8,830	144,039	1,585	71,379	1329	45,430
	Cohabiting	643	25,094	903	31,820	993	36,699	912	62,058	1017	42,646
	Married	557	81,249	829	77,774	897	100,531	3,959	402,554	3039	234,098
	Separated	184	19,515	274	18,437	306	20,994	869	66,900	689	28,578
Level of education	Low	7494	184,147	827	82,895	1934	86,642	3,681	398,535	842	70,193
	Medium	2374	73,041	2863	110,982	6638	165,832	2,361	149,762	3845	224,871
	High	2753	81,166	1846	37,040	2454	49,789	1,283	54,594	1387	55,688
Total		12,621	338,353	5536	230,916	11,026	302,263	7,325	602,891	6074	350,752

*Source* Authors’ calculations using data from the UK Household Longitudinal Study (UKHLS) for the UK, Trajectories and Origins (TeO) for France, and the German Socio-Economic Panel (SOEP) for Germany

**Table 4:** Number of events and person-months among the female descendants of immigrants and native women in the UK, France, and Germany by categories of covariates included in the analyses for employment exit

		First employment exit					
		UK		France		Germany	
		Events	Person months	Events	Person months	Events	Person months
Time since start of employment	0–1 year	1146	157,000	202	62,924	370	125,639
	1–3 years	1639	268,811	527	109,285	1459	211,496
	3–5 years	1241	229,380	360	91,974	1101	168,784
	5 + years	5776	1,358,361	1071	442,959	4504	664,677
Age at start of employment	< 20	7436	1,509,092	828	245,794	2057	315,332
	20–24	1992	422,172	1013	339,721	4281	705,453
	25–30	191	48,518	264	99,597	761	123,980
	30 +	183	33,770	55	22,029	335	25,830
Migration background	Natives	8273	1,733,129	603	241,198	6376	1,043,437
	2G Europe & Western	630	132,793				
	2G India	164	34,319				
	2G Pakistan	155	15,705				
	2G Bangladesh	102	10,169				
	2G Caribbean	251	52,656				
	2G Africa	227	34,783				
	2G North Africa			513	123,485		
	2G Sub-Saharan Africa			113	28,181		
	2G South-East Asia			99	31,658		
	2G Turkey			118	15,990		
	2G Southern Europe			556	213,150		
	2G Other Europe			158	53,480		
	2G Poland					212	31,863
	2G Russia/Kazakhstan					200	22,306
	2G Southern Europe					278	37,057
	2G Turkey					368	35,933
Birth cohort	1940–49	2139	389,892	44	20,909	371	100,506
	1950–59	2120	460,506	318	165,076	585	172,779
	1960–69	2050	574,010	748	291,027	1903	360,212
	1970–79	1618	394,273	764	190,336	2276	310,869
	1980–89	1161	159,063	283	39,713	1547	160,240
	1990+	714	35,809	3	81	752	65,989
Parity	No child	5797	1,214,174	847	318,629	3149	804,181
	One or more children	4005	799,378	1313	388,513	4285	366,415
Partnership status	Single	2760	691,303	456	181,101	2089	491,680
	Cohabiting	1225	255,913	396	117,198	1239	204,709
	Married	5039	876,787	1070	322,302	3555	382,868
	Separated	778	189,549	238	86,540	551	91,339
Level of education	Low	5231	964,362	478	116,401	1188	142,795
	Medium	2054	400,652	1,290	374,068	4656	733,287
	High	2517	648,539	392	216,673	1590	294,514
Total		9802	2,013,552	2160	707,142	7434	1,170,596

*Source* Authors’ calculations using data from the UK Household Longitudinal Study (UKHLS) for the UK, Trajectories and Origins (TeO) for France, and the German Socio-Economic Panel (SOEP) for Germany

**Table 5:** Hazard ratios (HR) of first employment entry, employment exit, and employment re-entry among immigrant women

		First employment entry			First employment exit			Employment re-entry		
		HR	Std. Err	Sig	HR	Std. Err	Sig	HR	Std. Err	Sig
Time since migration	0–1 year (ref)	1								
	1–3 years	0.238	0.011	***						
	3–5 years	0.304	0.015	***						
	5 + years	0.257	0.011	***						
Age at migration	15–19 (ref)	1								
	20–24	1.248	0.059	***						
	25+	1.586	0.077	***						
Time since start of employment	0–1 year (ref)				1					
	1–3 years				0.893	0.058				
	3–5 years				0.771	0.056	***			
	5 + years				0.483	0.033	***			
Age at start of employment	< 20 (ref)				1					
	20–24				0.840	0.080				
	25–30				0.780	0.076	*			
	30 +				0.627	0.062	***			
Time since employment exit	0–1 year (ref)							1		
	1–3 years							0.551	0.042	***
	3–5 years							0.433	0.044	***
	5 + years							0.309	0.029	***
Age at employment exit	< 25 (ref)							1		
	25–29							0.831	0.100	
	30–34							0.820	0.100	
	35 +							0.964	0.116	
Migrant origin	UK									
	1G Europe & Western (ref)	1			1			1		
	1G India	0.572	0.046	***	0.749	0.095	*	0.694	0.102	*
	1G Pakistan	0.143	0.019	***	1.806	0.315	**	0.422	0.108	**
	1G Bangladesh	0.149	0.023	***	2.673	0.514	***	0.456	0.124	**
	1G Caribbean	1.161	0.132		0.753	0.127		0.888	0.166	
	1G Africa	0.684	0.045	***	1.422	0.132	***	0.772	0.082	*
	France									
	1G North Africa	0.295	0.021	***	0.860	0.096				
	1G Sub-Saharan Africa	0.518	0.033	***	0.894	0.094				
	1G South-East Asia	0.502	0.038	***	0.838	0.103				
	1G Turkey	0.241	0.027	***	0.999	0.173				
	1G Southern Europe	0.888	0.069		0.405	0.057	***			
	1G Other Europe	0.569	0.038	***	0.799	0.089	*			
	Germany									
	1G Poland	0.911	0.062		1.584	0.151	***	0.964	0.108	
	1G Russia/Kazakhstan	0.882	0.055	*	1.623	0.145	***	0.771	0.081	*
	1G Southern Europe	0.813	0.070	*	1.285	0.155	*	0.896	0.127	
	1G Turkey	0.389	0.037	***	1.921	0.234	***	0.786	0.111	
Migration cohort	1956–1989 (ref)	1			1			1		
	1990–1999	0.966	0.041		1.641	0.106	***	1.437	0.138	***
	2000 +	1.024	0.044		2.447	0.165	***	1.664	0.153	***
Parity	No child (ref)	1			1			1		
	One or more children	0.617	0.024	***	1.320	0.079	**	0.832	0.072	*
Partnership status	Single (ref)	1			1			1		
	Cohabiting	1.378	0.078	***	1.276	0.121	*	1.070	0.150	
	Married	1.103	0.049	*	1.265	0.096	**	1.035	0.117	
	Separated	1.285	0.082	***	1.247	0.122	*	1.210	0.173	
Level of education	Low (ref)	1			1			1		
	Medium	1.233	0.047	***	0.904	0.050		1.218	0.097	*
	High	1.984	0.081	***	0.841	0.049	**	1.410	0.107	***
Constant		0.041	0.003	***	0.004	0.001	***	0.028	0.004	***

*Source*: Authors’ calculations using data from the UK Household Longitudinal Study (UKHLS) for the UK, Trajectories and Origins (TeO) for France, and the German Socio-Economic Panel (SOEP) for Germany. *Note ** p < 0.05; ** p < 0.01; *** p < 0.001

**Table 6:** Hazard ratios (HR) of first employment entry, employment exit, and employment re-entry among the female descendants of immigrants and native women

		First employment entry			First employment exit			Employment re-entry		
		HR	Std. Err	Sig	HR	Std. Err	Sig	HR	Std. Err	Sig
Time since leaving education	0–1 year (ref)									
	1–3 years	0.424	0.007	***						
	3–5 years	0.209	0.006	***						
	5 + years	0.091	0.003	***						
Age at leaving education	< 20 (ref)									
	20 +	1.007	0.017							
Time since start of employment	0–1 year (ref)									
	1–3 years				1.254	0.037	***			
	3–5 years				1.063	0.034				
	5 + years				0.721	0.022	***			
Age at start of employment	< 20 (ref)									
	20–24				0.927	0.018	***			
	25–30				0.863	0.030	***			
	30 +				1.072	0.049				
Time since employment exit	0–1 year (ref)									
	1–3 years							0.589	0.014	***
	3–5 years							0.464	0.014	***
	5 + years							0.439	0.011	***
Age at employment exit	< 25 (ref)									
	25–29							1.080	0.032	**
	30–34							1.079	0.036	*
	35 +							1.263	0.043	***
Migration background	UK									
	UK natives (ref)									
	2G Europe & Western	0.860	0.031	***	1.085	0.045	*	0.965	0.046	
	2G India	0.679	0.045	***	0.957	0.076		0.990	0.086	
	2G Pakistan	0.491	0.034	***	1.458	0.119	***	0.497	0.053	***
	2G Bangladesh	0.509	0.041	***	1.326	0.133	**	0.596	0.075	***
	2G Caribbean	0.689	0.039	***	1.165	0.075	*	0.829	0.061	*
	2G Africa	0.602	0.034	***	1.268	0.086	***	0.925	0.073	
	France									
	French natives	0.954	0.028		0.544	0.024	***			
	2G North Africa	0.601	0.019	***	0.815	0.038	***			
	2G Sub-Saharan Africa	0.482	0.027	***	0.744	0.071	**			
	2G South-East Asia	0.632	0.037	***	0.631	0.064	***			
	2G Turkey	0.605	0.042	***	0.999	0.094				
	2G Southern Europe	1.004	0.030		0.547	0.025	***			
	2G Other Europe	0.769	0.041	***	0.681	0.055	***			
	Germany									
	German natives	0.983	0.015		1.305	0.027	***	0.927	0.020	**
	2G Poland	1.107	0.064		1.406	0.099	***	0.995	0.076	
	2G Russia/Kazakhstan	0.992	0.056		1.109	0.082		0.957	0.083	
	2G Southern Europe	1.047	0.051	**	1.333	0.083	***	1.159	0.080	*
	2G Turkey	0.859	0.038		1.434	0.079	***	0.955	0.061	
Birth cohort	1940–49 (ref)									
	1950–59	0.908	0.023	***	0.822	0.022	***	1.167	0.039	**
	1960–69	0.742	0.017	***	0.893	0.023	***	1.448	0.046	***
	1970–79	0.699	0.017	***	1.224	0.032	**	1.789	0.058	***
	1980–89	0.669	0.017	***	1.963	0.058	***	2.271	0.083	***
	1990+	0.565	0.015	***	3.562	0.129	***	2.718	0.129	***
Parity	No child (ref)									
	One or more children	0.624	0.018	***	1.621	0.030	***	0.699	0.017	***
Partnership status	Single (ref)									
	Cohabiting	1.072	0.024	**	1.449	0.036	***	0.986	0.031	
	Married	0.821	0.023	***	2.061	0.047	***	0.890	0.026	***
	Separated	1.083	0.042	*	1.412	0.045	***	1.151	0.040	***
Level of education	Low (ref)									
	Medium	1.080	0.016	***	0.873	0.017	***	1.427	0.033	***
	High	1.174	0.022	***	0.668	0.015	***	1.779	0.044	***
Constant		0.117	0.002	***	0.003	0.000	***	0.016	0.001	***

*Source*: Authors’ calculations using data from the UK Household Longitudinal Study (UKHLS) for the UK, Trajectories and Origins (TeO) for France, and the German Socio-Economic Panel (SOEP) for Germany. *Note ** p < 0.05; ** p < 0.01; *** p < 0.001

**Table 7:** Hazard ratios (HR) of first employment entry, employment exit, and employment re-entry among immigrant women, interactions between country, origin, and parity

		First employment entry			First employment exit			Employment re-entry		
		HR	Std. Err	Sig	HR	Std. Err	Sig	HR	Std. Err	Sig
Time since migration	0–1 year (ref)									
	1–3 years	0.241	0.011	***						
	3–5 years	0.312	0.015	***						
	5 + years	0.272	0.012	***						
Age at migration	15–19 (ref)									
	20–24	1.204	0.058	***						
	25 +	1.505	0.073	***						
Time since start of employment	0–1 year (ref)									
	1–3 years				0.901	0.059				
	3–5 years				0.782	0.057	**			
	5 + years				0.494	0.033	***			
Age at start of employment	< 19 (ref)									
	20–24				0.845	0.080				
	25–30				0.791	0.077	*			
	30 +				0.637	0.063	***			
Time since employment exit	0–1 year (ref)									
	1–3 years							0.554	0.042	***
	3–5 years							0.434	0.045	***
	5 + years							0.312	0.029	***
Age at employment exit	< 24 (ref)									
	25–29							0.858	0.104	
	30–34							0.849	0.105	
	35 +							0.984	0.119	
Migrant origin x parity	UK									
	No child x 1G Europe & Western (ref)									
	No child x 1G India	0.612	0.063	***	1.487	0.275	*	0.964	0.250	
	No child x 1G Pakistan	0.169	0.033	***	1.579	0.498		0.769	0.328	
	No child x 1G Bangladesh	0.169	0.043	***	2.988	1.034	**	0.385	0.276	
	No child x 1G Caribbean	1.008	0.146		0.892	0.239		0.614	0.284	
	No child x 1G Africa	0.640	0.053	***	1.542	0.219	**	0.599	0.123	*
	France									
	No child x 1G North Africa	0.377	0.035	***	0.633	0.144	*			
	No child x 1G Sub-Saharan Africa	0.312	0.031	***	0.764	0.185				
	No child x 1G South-East Asia	0.497	0.049	***	0.600	0.142	*			
	No child x 1G Turkey	0.217	0.043	***	0.554	0.324				
	No child x 1G Southern Europe	1.189	0.114		0.416	0.118	**			
	No child x 1G Other Europe	0.628	0.052	***	0.718	0.135				
	Germany									
	No child x 1G Poland	0.688	0.064	***	1.263	0.200		0.715	0.153	
	No child x 1G Russia/Kazakhstan	0.507	0.049	***	1.633	0.257	**	0.455	0.113	**
	No child x 1G Southern Europe	0.731	0.077	**	1.020	0.204		0.835	0.215	
	No child x 1G Turkey	0.341	0.048	***	2.699	0.578	***	0.504	0.179	
	UK									
	Child x 1G Europe & Western	0.457	0.047	***	1.258	0.165		0.647	0.098	**
	Child x 1G India	0.281	0.032	***	0.684	0.120	*	0.424	0.083	***
	Child x 1G Pakistan	0.071	0.012	***	2.453	0.523	***	0.233	0.077	***
	Child x 1G Bangladesh	0.078	0.015	***	3.318	0.782	***	0.329	0.100	***
	Child x 1G Caribbean	0.705	0.123	*	0.880	0.190		0.662	0.145	
	Child x 1G Africa	0.380	0.035	***	1.742	0.216	***	0.567	0.083	***
	France									
	Child x 1G North Africa	0.140	0.013	***	1.194	0.165				
	Child x 1G Sub-Saharan Africa	0.377	0.029	***	1.176	0.150				
	Child x 1G South-East Asia	0.275	0.029	***	1.197	0.181				
	Child x 1G Turkey	0.141	0.019	***	1.383	0.266				
	Child x 1G Southern Europe	0.361	0.041	***	0.519	0.088	***			
	Child x 1G Other Europe	0.276	0.026	***	1.067	0.152				
	Germany									
	Child x 1G Poland	0.653	0.059	***	2.230	0.274	***	0.717	0.108	*
	Child x 1G Russia/Kazakhstan	0.671	0.051	***	2.084	0.242	***	0.576	0.083	***
	Child x 1G Southern Europe	0.498	0.067	***	1.854	0.293	***	0.626	0.115	*
	Child x 1G Turkey	0.232	0.029	***	2.250	0.342	***	0.583	0.104	**
Migration cohort	1956–1989 (ref)									
	1990–1999	0.955	0.040		1.663	0.108	***	1.418	0.136	***
	2000 +	1.014	0.044		2.445	0.165	***	1.633	0.150	***
Partnership status	Single (ref)									
	Cohabiting	1.299	0.074	***	1.279	0.122	*	1.052	0.149	
	Married	1.042	0.046		1.236	0.095	**	1.025	0.117	
	Separated	1.194	0.077	**	1.222	0.120	*	1.185	0.170	
Level of education	Low (ref)									
	Medium	1.225	0.047	***	0.902	0.050		1.197	0.095	*
	High	2.044	0.084	***	0.843	0.050	**	1.406	0.107	***
Constant		0.046	0.003	***	0.004	0.001	***	0.033	0.006	***

*Source*: Authors’ calculations using data from the UK Household Longitudinal Study (UKHLS) for the UK, Trajectories and Origins (TeO) for France, and the German Socio-Economic Panel (SOEP) for Germany. *Note ** p < 0.05; ** p < 0.01; *** p < 0.001

**Table 8:** Hazard ratios (HR) of first employment entry, employment exit, and employment re-entry among the female descendants of immigrants and native women, interactions between country, origin, and parity

		First employment entry			First employment exit			Employment re-entry		
		HR	Std. Err	Sig	HR	Std. Err	Sig	HR	Std. Err	Sig
Time since leaving education	0–1 year (ref)									
	1–3 years	0.423	0.007	***						
	3–5 years	0.208	0.006	***						
	5 + years	0.094	0.003	***						
Age at leaving education	< 19 (ref)									
	20 +	1.010	0.017							
Time since start of employment	0–1 year (ref)									
	1–3 years				1.267	0.037	***			
	3–5 years				1.065	0.034	*			
	5 + years				0.743	0.023	***			
Age at start of employment	< 19 (ref)									
	20–24				0.903	0.017	***			
	25–30				0.802	0.028	***			
	30 +				1.023	0.047				
Time since employment exit	0–1 year (ref)									
	1–3 years							0.589	0.014	***
	3–5 years							0.466	0.014	***
	5 + years							0.442	0.011	***
Age at employment exit	< 24 (ref)									
	25–29							1.088	0.032	**
	30–34							1.077	0.036	*
	35 +							1.257	0.043	***
Migration background x parity	UK									
	No child x UK natives (ref)									
	No child x 2G Europe & Western	0.857	0.032	***	1.088	0.058		0.855	0.071	
	No child x 2G India	0.664	0.045	***	1.090	0.108		1.205	0.156	
	No child x 2G Pakistan	0.482	0.034	***	1.309	0.131	**	0.785	0.112	
	No child x 2G Bangladesh	0.511	0.042	***	1.338	0.159	*	0.849	0.132	
	No child x 2G Caribbean	0.677	0.040	***	1.168	0.104		0.857	0.104	
	No child x 2G Africa	0.593	0.035	***	1.191	0.099	*	0.954	0.108	
	France									
	No child x French natives	0.947	0.029		0.495	0.036	***			
	No child x 2G North Africa	0.578	0.020	***	0.798	0.055	**			
	No child x 2G Sub-Saharan Africa	0.405	0.025	***	0.818	0.113				
	No child x 2G South-East Asia	0.591	0.037	***	0.685	0.099	**			
	No child x 2G Turkey	0.594	0.044	***	0.820	0.127				
	No child x 2G Southern Europe	0.992	0.031		0.545	0.038	***			
	No child x 2G Other Europe	0.760	0.043	***	0.619	0.082	***			
	Germany									
	No child x German natives	0.958	0.015	**	0.731	0.020	***	0.831	0.030	***
	No child x 2G Poland	1.104	0.066		0.769	0.081	*	1.021	0.131	
	No child x 2G Russia/Kazakhstan	0.980	0.059		0.580	0.065	***	0.696	0.112	*
	No child x 2G Southern Europe	1.015	0.051		0.987	0.081		1.096	0.112	
	No child x 2G Turkey	0.838	0.039	***	1.003	0.073		1.004	0.095	
	UK									
	Child x UK natives	0.423	0.024	***	1.031	0.026		0.661	0.020	***
	Child x 2G Europe & Western	0.361	0.079	***	1.084	0.072		0.679	0.042	***
	Child x 2G India	0.583	0.239		0.820	0.108		0.567	0.068	***
	Child x 2G Pakistan	0.308	0.098	***	1.524	0.214	**	0.220	0.036	***
	Child x 2G Bangladesh	0.178	0.089	**	1.106	0.207		0.244	0.053	***
	Child x 2G Caribbean	0.427	0.069	***	1.265	0.117	*	0.536	0.051	***
	Child x 2G Africa	0.377	0.098	***	1.265	0.147	*	0.585	0.066	***
	France									
	Child x French natives	0.541	0.043	***	0.693	0.037	***			
	Child x 2G North Africa	0.419	0.033	***	0.968	0.061				
	Child x 2G Sub-Saharan Africa	0.607	0.065	***	0.825	0.108				
	Child x 2G South-East Asia	0.575	0.089	***	0.684	0.098	**			
	Child x 2G Turkey	0.370	0.065	***	1.334	0.156	*			
	Child x 2G Southern Europe	0.594	0.049	***	0.653	0.038	***			
	Child x 2G Other Europe	0.451	0.064	***	0.859	0.088				
	Germany									
	Child x German natives	0.656	0.026	***	2.483	0.066	***	0.647	0.021	***
	Child x 2G Poland	0.584	0.122	*	2.918	0.274	***	0.652	0.062	***
	Child x 2G Russia/Kazakhstan	0.585	0.092	**	2.306	0.222	***	0.735	0.076	**
	Child x 2G Southern Europe	0.838	0.157		1.958	0.183	***	0.793	0.076	*
	Child x 2G Turkey	0.574	0.081	***	2.296	0.186	***	0.603	0.052	***
Birth cohort	1940–49 (ref)									
	1950–59	0.904	0.023	***	0.822	0.022	***	1.167	0.039	***
	1960–69	0.735	0.017	***	0.916	0.023	**	1.447	0.046	***
	1970–79	0.694	0.016	***	1.256	0.033	**	1.794	0.058	***
	1980–89	0.667	0.016	***	2.028	0.059	***	2.290	0.084	***
	1990 +	0.562	0.015	***	4.153	0.151	***	2.739	0.131	***
Partnership status	Single (ref)									
	Cohabiting	1.071	0.024	**	1.449	0.037	***	0.990	0.032	
	Married	0.824	0.023	***	2.051	0.047	***	0.891	0.026	***
	Separated	1.087	0.042	*	1.412	0.045	***	1.154	0.040	***
Level of education	Low (ref)									
	Medium	1.081	0.016	***	0.879	0.017	***	1.420	0.033	***
	High	1.171	0.022	***	0.691	0.015	***	1.786	0.044	***
Constant		0.119	0.002	***	0.004	0.000	***	0.017	0.001	***

*Source*: Authors’ calculations using data from the UK Household Longitudinal Study (UKHLS) for the UK, Trajectories and Origins (TeO) for France, and the German Socio-Economic Panel (SOEP) for Germany. *Note ** p < 0.05; ** p < 0.01; *** p < 0.001

**Table 9:** List of countries included in the broader migrant origin categories

Destination country	Region of origin	Origin countries
UK	Europe & Western countries	France, Germany, Italy, Ireland, Spain, Poland, Cyprus, Turkey, Portugal, Albania, Armenia, Austria, Belarus, Belgium, Bosnia and Herzegovina, Bulgaria, Channel Islands, Australia, Czech Republic, Denmark, Finland, Georgia, Gibraltar, Greece, Hungary, Jersey, Kosovo, Latvia, Lithuania, Malta, Moldova, Norway, Portugal, Romania, Russia, Serbia, Slovakia, Slovenia, Sweden, Switzerland, the Netherlands, Ukraine, Yugoslavia, New Zealand, Canada, USA
	Caribbean countries	Jamaica, Anguilla, Antigua, Bahamas, Barbados, Cuba, Dominica, Dominican Republic, Guadeloupe, Grenada, Guyana, Haiti, Montserrat, Nevis, St Lucia, St Vincent and the Grenadines, Trinidad and Tobago
	African countries	Kenya, Ghana, Nigeria, Uganda, Algeria, Angola, Benin, Botswana, Burkina Faso, Burundi, Cameroon, Cape Verde, Zaire, Democratic Republic of Congo, Djibouti, Egypt, Eritrea, Ethiopia, Gabon, Gambia, Guinea, Guinea-Bissau, Ivory Coast, Liberia, Libya, Madagascar, Malawi, Mauritius, Morocco, Mozambique, Namibia, Rwanda, Senegal, Seychelles, Sierra Leone, Somalia, Sudan, Swaziland, Tanzania, Togo, Tunisia, Zambia, Zimbabwe
France	North Africa	Algeria, Morocco, Tunisia
	Sub-Saharan Africa	Senegal, Mauritania, the Gambia, Guinea-Bissau, Guinea, Mali, Burkina Faso, Niger, Chad, Ivory Coast, Ghana, Togo, Benin, Nigeria, Cameroon, Central African Republic, Gabon, Republic of the Congo, DRC, Equatorial Guinea
	South-East Asia	Vietnam, Laos, Cambodia
	Southern Europe	Portugal, Italy, Spain, Greece, Cyprus, Malta
	Other Europe	Austria, Germany, Luxembourg, Denmark, Sweden, Finland, UK, Ireland, Belgium, the Netherlands, Bulgaria, Estonia, Hungary, Latvia, Lithuania, Poland, Czech Republic, Romania, Slovenia, Slovakia
Germany	Southern Europe	Spain, Italy, Portugal, Greece

## Footnotes

1 Not including more recent sending countries such as East Asian countries.

2 Immigrants are born outside the destination countries whereas the descendants of immigrants are born in the destination countries to at least one immigrant parent.

3 Much of the available evidence in the UK focuses on differences by race/ethnicity as opposed to country of birth. Although this paper does not explore the role of ethnicity, we summarise the available evidence on ethnic differences in employment and fertility.

4 The TeO2 dataset has become available for public use after the completion of this study. Future studies should consider using this dataset.

5 See Note 1 in the Appendix.

6 Appendix Table 9 shows which countries are included in each origin group. Although some of the origin countries included in the origin groups defined across counties overlap, we cannot separate out the exact same origin groups or origin countries in the UK, France, and Germany due to sample size limitations. For example, we can study individuals from Southern Europe in France whilst in the UK they are grouped into the European/Western category because the data does not allow us to divide European/Western individuals into smaller origin groups.

7 The information on higher-order employment entries is limited in the French data.
